# Comparison on Bioactivities and Characteristics of Polysaccharides From Four Varieties of *Gastrodia elata* Blume

**DOI:** 10.3389/fchem.2022.956724

**Published:** 2022-07-22

**Authors:** Ning Ji, Peng Liu, Ni Zhang, Shengyan Yang, Mingsheng Zhang

**Affiliations:** ^1^ Key Laboratory of Plant Resource Conservation and Germplasm Innovation in Mountainous Region (Ministry of Education), Collaborative Innovation Center for Mountain Ecology & Agro-Bioengineering(CICMEAB), College of Life Sciences/Institute of Agro-bioengineering, Guizhou University, Guiyang, China; ^2^ Food and Pharmaceutical Engineering Institute, Guiyang University, Guiyang, China; ^3^ Dejiang Lvtong Gastrodia elata Development Co., Ltd., Tongren, China

**Keywords:** *Gastrodia elata* blume., polysaccharide, variety, physicochemical property, biological activity

## Abstract

The composition, physicochemical properties, *in vitro* biological activity, and hypoglycemic activity exhibited by polysaccharides from four varieties of *G. elata* were investigated in this study; the four extracted GaE polysaccharides were termed as GaE-B (*G. elata Bl. f. glauca S. chow* polysaccharides), GaE-R (*G. elata Bl. f. elata* polysaccharides), GaE-Hyb (hybridization of *G. elata Bl. f. glauca S. chow* and *G. elata Bl. f. elata* polysaccharides), and GaE-G (*G. elata Bl. f. viridis Makino* polysaccharides). As revealed by the results, the GaE polysaccharides were found with the same monosaccharide composition, primarily including glucose, whereas the content of each variety was significantly different. In addition, different degrees of differences were found in the *in vitro* antioxidant and hypoglycemic activity, molecular weight, yield, and chemical composition exhibited by the abovementioned varieties. However, GaE-B and GaE-Hyb were found with similar physical properties, chemical composition, and antioxidant and hypoglycemic activity. GaE-R had the lowest yield, total sugar content, and molecular weight, whereas it involved higher xylose, binding protein, and polyphenols as well as higher antioxidant and hypoglycemic activity. In contrast, GaE-G was found with the highest yield, total sugar content, and molecular weight, whereas it contained the lowest xylose, binding protein, and polyphenols, as well as the weakest antioxidant and hypoglycemic activity. In brief, the polysaccharide of *G. elata*, a plant resource for homology of medicine and food, could more significantly enhance the biological activity of *G. elata* as it was released in the process of decocting and stewing. To be specific, the assessment of polysaccharide activity alone suggested that GaE-R was the best.

## 1 Introduction

Over the past few years, increasing studies have been conducted on various physiological and biochemical characteristics of plant polysaccharides. Plant polysaccharides, a natural polymer, have less irritation to the body when exerting their pharmacological properties. The bioactivities of plant polysaccharides are largely manifested in anti-inflammatory, antioxidation, anti-tumor, anti-virus, anti-diabetes, immune regulation, etc. (Yue et al., 2017; Chen L. et al., 2018; Chen Y. et al., 2018; Fan et al., 2018; Ma et al., 2018). The above mentioned characteristics of plant polysaccharides are capable of facilitating the development and utilization of a range of health foods and medicines. The physical and chemical properties exhibited by polysaccharides take on a critical significance to their physiological and functional properties. In particular, the chemical composition, molecular weight and distribution, monosaccharide composition, and functional group differences in polysaccharides have effects on the physiological and biochemical properties exhibited by polysaccharides (Nie et al., 2017). Accordingly, studying the physical and chemical properties exhibited by plant polysaccharides can assess the biological activity and application value of the abovementioned plants from one side.

*G. eleta* (Orchidaceae) refers to an orchid plant, a fully mycoheterotrophic plant, forming a symbiotic relationship with Armillaria mellea (Cha and Igarashi, 1995) during its growth. It is a traditional Chinese herbal medicine (Yuan et al., 2018). It has been recorded in ancient China, and its dry tubers are commonly used as medicine (Chinese Pharmacopoeia Commission, 2015). It is currently planted in East Asian nations (e.g., China, Japan, South Korea, and India) (Lee et al., 2014). *G. elata* is largely adopted to treat dizziness, cancer, depression, epilepsy, convulsion, inflammation, liver protection, anti-aging, neuroprotection, and other diseases (Chen J. et al., 2016; Chen L. et al., 2016; Zhan et al., 2016; Kim et al., 2017; Lin et al., 2018; Wang et al., 2018; Zhang et al., 2018; Ma et al., 2020). The major medicinal components of *G. elata* are gastrodin p-hydroxybenzyl alcohol and p-hydroxybenzaldehyde (Chinese Pharmacopoeia Commission, 2015; Li et al., 2019). Second, *G. elata* also contains polyphenols, polysaccharides, organic acids, and other active components (Ojemann et al., 2006). In China, different varieties of *G. elata* have been employed for cultivation under forest (Zhan et al., 2016). The major difference of *G. elata* varieties refers to the not consistent color of the stem after the process of bolting and flowering. At present, *G. elata Bl. f. glauca S. chow*, *G. elata Bl. f. elata*, *G. elata Bl. f. viridis Makino*, and Hybrid *G. elata Bl.* (Hybridization of *G. elata Bl. f. glauca S. chow* and *G. elata Bl. f. elata*) have been primarily planted in Guizhou, China, whereas the adaptability and growth trend of the varieties of *G. elata* are different, thus resulting in the differences of biological activities (Zhan et al., 2016). Over the past few years, *G. elata* polysaccharides have aroused wide attention for their various physiological activities and functions (e.g., anticancer, bioactivity on gut microbiota, anti-aging, immunomodulation, neuroprotection, and prevention of cardiovascular and cerebrovascular diseases, as well as other activities) (Chen et al., 2011; Kim et al., 2012; ; Chen L. et al., 2016; Zhou et al., 2017; Chen L. et al., 2018; Huo et al., 2021). As revealed by the abovementioned activity results, *G. elata* polysaccharides play a vital role in the whole pharmacological activity of *G. elata*. Although a considerable number of reports on the structure, composition, and activity of *G. elata* polysaccharide have been conducted, the focus has been placed on a single variety, and there have been rare relevant research reports on the differences in physical properties, chemical properties, and biological activities between different varieties.

In this study, the selection of varieties in the cultivation process has been controversial in the experimental *G. elata* planting area, Dejiang County, Guizhou Province, China. At this stage, *G. elata* polysaccharides have also been recognized as one of the key natural compounds in the study of active components of *G. elata*. Thus, this study aimed to compare the physicochemical properties and biological activities of non-starch polysaccharides in different varieties of *G. elata* from the perspective of *G. elata* polysaccharides, so as assess the differences of polysaccharides between different varieties of *G. elata*. Indeed, the differences of polysaccharides do not represent the differences of the overall quality of *G. elata*, and the differences of varieties cannot represent the differences between regions. Impacted by the different natural conditions (e.g., weather, altitude, soil, and rainfall in the planting area), the physical and chemical indicators of *G. elata* planted will be different. As an ancient saying in China goes, “A side water and soil raises a side person” (i.e., the unique features of a local environment always give special characteristics to its inhabitants). We consider that this ancient saying is also suitable for plants. The difference of polysaccharide in different varieties of *G. elata* planted in our experimental area does not represent the variety difference in the entire Chinese region, whereas they can serve as an assessment of variety selection and application value of locally planted *G. elata*.

## 2 Materials and Methods

### 2.1 Materials

The fresh tubers of four varieties of *G. elata* (*G. elata* Bl. f. glauca S. chow, *G. elata* Bl. f. elata, Hybridization of *G. elata* Bl. f. glauca S. chow, and *G. elata* Bl. f. elata (*G. elata Bl. f. elata* was the female parent and *G. elata Bl. f. glauca S. chow* was the male parent) and G. elata Bl. f. viridis Makino, the extracted polysaccharides were termed GaE-B, GaE-R, GaE-Hyb, and GaE-G) were collected from the planting base of Dejiang Lvtong *G. elata* Blume. Development Co., Ltd. (108°8′49.36″E, 28°8′1.12″N, and the altitude was 1091.32 m, the annual average temperature was 16.0°C, and the annual average rainfall was 1237 mm), Guizhou Province, China, and they were planted at the same time. The fresh *G. elata* tubers were washed and cut into slices. Subsequently, the tubers were dried at 50°C, and the moisture content was controlled in a range of 3%–5% (determination of moisture content by the constant weight method). Next, the tubers were crushed into powder and passed through a 60-mesh sieve. The sieved powder was stored in sealed bags for standby. The treated powder was soaked in petroleum ether (material liquid ratio was 1:3), and the mixture was stirred continuously with a mechanical stirrer (boiling point range: 60–90°C). The petroleum ether was replaced every 8 h for a total of 3 times. The powder soaked in petroleum ether was dried and then soaked in 95% ethanol (v/v) for 3 times (material liquid ratio was 1:3). In the soaking period, a mechanical agitator was used for continuous stirring. After the filtration, the sample was dried at 40°C for later use (termed GaEs). Monosaccharides, pigments, lipids, oligosaccharides, and small molecular impurities could be removed through soaking in petroleum ether and ethanol.

The reagents applied in this study consisted of ascorbic acid (Vc), acarbose, bovine serum albumin (BSA), 1-pheny-3-methyl-5-pyrazolone (PMP), trifluoroacetic acid (TFA), 3-phenylphenol, 2,2-diphenyl-1-picrylhydrazyl (DPPH), phenazine methosulfate, β-nicotinamide adenine dinucleotide (NADH), 2,2-diphenyl-1-picrylhydrazyl (DPPH), nitrotetrazolium blue chloride (NBT), 2,2′-azino-bis(3-ethylbenzothiazoline-6-sulfonic acid) diammonium salt (ABTS), 3-(2-pyridyl)-5,6-bis-(4-phenylsulfonicacid)-1,2,4-triazine monosodium salt, α-glucosidase (50 U/mg, BR), α-amylase (50 U/mg, BR), salicylic acid, ethylenediaminetetraacetic acid (EDTA), 4-nitrophenyl α-D-galacto-pyran-oside (PNGP), 3,5-dinitrosalicylic acid, and standard dextran (10, 40, 70, 100, 150, 270, and 410 Kda). Standard monosaccharides (Purity ≥ 99%), consisting of D-mannose (Man), L-rhamnose (Rha), D-glucuronic acid (GlcA), D-galacturonic acid (GalA), D-lactose (Lac), D-glucose (Glc), D-galactose (Gal), D-xylose (Xyl), and L-arabinose (Ara) originated from Shanghai Macklin Biochemical Technology Co., Ltd. (Shanghai, China), and other used chemical reagents were of analytical grade and purchased locally. Dialysis bags (molecular weight cut-off, 8000–14,000 Da) were provided by Union Carbide Co., Ltd. (United States).

### 2.2 Polysaccharide Extraction and Isolation

The GaE polysaccharide was extracted using the water extraction method (Yan et al., 2018) and modified slightly. 25.0 g of pretreated *G. elata* powder was poured into 500 ml distilled water and extracted in a 90°C water bath for 4 h. After the extraction, the mixture was subjected to vacuum decompression filtration. Next, the filter residue was extracted and filtered using the same method, and the two filtrates were combined. Subsequently, the filtrate was concentrated to 1/5 of the original volume using a rotary evaporator (RV10, IKA, Germany) in a 50°C water bath. The concentrated solution was precipitated with anhydrous ethanol (ethanol: concentrated solution = 5:1, v/v) and then placed at 4°C for 12 h, so it could be sufficiently precipitated. Afterward, the mixed solution was centrifuged at 6500 rpm for 10 min, and the precipitates of combining were washed 3 times with anhydrous ethanol, anhydrous ether, and acetone. In the following, the precipitate was dissolved with a small amount of distilled water. The following used sevag reagent (n-butanol: trichloromethane = 1:4, v/v) to remove the associated protein (Sevag et al., 1938). The aqueous layer after the removal of the protein was placed into dialysis bag (molecular weight cut-off 8000–14,000 Da) and then dialyzed for 72 h in flowing distilled water. After the dialysis, the solution was placed in a vacuum freeze dryer (BenchTop Pro-EL, Virtis, United States) for vacuum freeze-drying, all extraction steps were repeated 3 times, and the average value was taken. The partially purified *G. elata* polysaccharides were termed below: GaE-B (*G. elata Bl. f. glauca S. chow polysaccharides*), GaE-R (*G. elata Bl. f. elata polysaccharides*), GaE-Hyb (Hybridization of *G. elata Bl. f. glauca S. chow and G. elata Bl. f. elata polysaccharides*), and GaE-G (*G. elata Bl. f. viridis Makino polysaccharides*). The yield (%) of *G. elata* polysaccharides was obtained by:

$$Yield\left(\%,w/w\right)=\frac{weight\;of\;dried\;polysaccharides\left(g\right)}{weight\;of\;extracted\;GaEs\;powder\left(g\right)}\times 100.$$

### 2.3 Chemical Composition Determination

The total sugar content of the GaE polysaccharide was obtained using the phenol–sulfuric acid method (DuBois et al., 1956) with D-glucose as the standard. The protein content was obtained using the Bradford method (Bradford, 1976) with bovine serum albumin (BSA) as the standard, uronic acid content was obtained using the m-hydroxybiphenyl method (Blumenkrantz and Asboe-Hansen, 1973), total phenol content was obtained using the Folin–Ciocalteu method (Chen et al., 2014) with gallic acid as the standard, and sulfate was obtained using the barium chloride–gelatin method (Tabatabai, 1974), and total flavonoids were obtained by colorimetry (Zhishen et al., 1999) with rutin as the standard. All indicators were measured 3 times.

### 2.4 Determination of Molecular Weight

The molecular weight (Mw) of the GaE polysaccharide was measured using the high-performance gel permeation chromatography (HPGPC) method in accordance with a previous report (He et al., 2018). A Shimadzu LC-2010A HPLC system (Shimadzu Co., Ltd., Japan) was used as the experimental instrument, the detector was refractive index detector (RID-10A, Shimadzu Co., Ltd., Japan), and TSK-Gel G4000 SWXL column (7.8 mm × 300 mm, Tosoh Biosep, Japan) was adopted as the chromatographic column. The experimental methods are elucidated below. The sample solution (1.5 mg/ml) was first filtered with a pore size of 45 μm aqueous membrane, and 25 μL was injected into the chromatographic column each time, 0.02 mol/L KH_2_PO_4_ served as the mobile phase, and the elution process was performed at a flow rate of 0.7 ml/min. The chromatographic column temperature was maintained at 35°C. The same method for dextran was employed as a standard (Macklin Biochemical Technology Co., Ltd., Shanghai, China) with different molecular weights (10, 40, 70, 100, 150, 270, and 410 kDa).

### 2.5 Monosaccharide Composition Analysis

First, 10 mg of the GaE polysaccharide sample was dissolved in 4 ml of trifluoroacetic acid solution (4 mol/L), sealed and hydrolyzed at 120°C for 4 h. Subsequently, PMP derivatization was performed using the method of Nie et al. (2017). The derivatized samples were investigated through HPLC with the use of a Shimadzu LC-2010A HPLC system (Shimadzu Co., Ltd., Japan) and a detector: UV analysis detector. A Wonda Sil C18-WR (4.6 mm × 150 mm×5 μm, Shimadzu Co., Ltd., Japan) was used as the chromatographic column. The specific method is elucidated below: the mobile phase was a mixture of 80% of 0.06 M phosphate buffer (pH 6.5) and 20% acetonitrile, at a flow rate of 0.8 ml/min with a UV detection wavelength of 250 nm, an injection volume of 10 μL and at a column temperature of 28°C. The monosaccharide standards were studied using the same method.

### 2.6 Fourier-Transform Infrared Spectroscopy Analysis

First, 1.5 mg of the dried GaE polysaccharide powder and 150 mg of the dried potassium bromide powder were mixed, ground, and pressed into granules. Subsequently, the granules were scanned in the range of 4000–400 cm^−1^ through Fourier transform infrared (FT-IR) spectroscopy (Nicolet iS5, Thermo Fisher Scientific Co., Ltd., United States).

### 2.7 Thermal Analysis

The GaE polysaccharide sample was fixed in a platinum crucible, and the thermal characteristics were studied with a thermogravimetric analyzer TGA, DTG-60A (Shimadzu Co., Ltd., Japan). The specific experimental operation is elucidated below. The nitrogen gas was maintained at a flow rate of 50 ml/min in the measurement. The initial temperature ranged from 30°C to 800°C, and the temperature increased at 10°C/min.

### 2.8 Measurement of Zeta Potential and Particle Size

The sample of the GaE polysaccharide was dissolved in distilled water to prepare the solution (1 mg/ml). A Phase Analysis Light Scattering analyzer (90 Plus PALS, Brookhaven Instruments Co., Ltd., United States) was employed to determine the zeta potential and particle size of the GaE polysaccharide. The respective sample was measured 3 times, and the average value was taken.

### 2.9 Scanning Electron Microscopy Analysis

The dried *G. elata* polysaccharide samples were sprayed with gold–palladium alloy and scanned using a scanning electron microscope (Gemini 300, ZEISS, Germany). Scanning electron microscopy (SEM) images were captured at a magnification of 300× and 1500× based on a voltage of 2 kV acceleration under high vacuum conditions.

### 2.10 Determination of Antioxidant Activities

#### 2.10.1 Superoxide Radical Scavenging Activity

The superoxide radical scavenging activity of the GaE polysaccharide was assessed using a method described previously (Bi et al., 2013) with slight modifications. The GaE polysaccharide samples of different concentrations (0.25–5.00 mg/L) consisted of 100 μL and 100 μL phenazine methosulfate (PMS) solutions (120 μmol/L), nitrotetrazolium blue chloride (NBT) solution (300 μmol/L), and β-nicotinamide adenine dinucleotide (NADH) solution (936 μmol/L). A 100 μmol/L phosphate buffer (PBS, pH 7.4) was added. Subsequently, the mixture was stirred and placed in the dark environment at 25°C for reaction for 5 min. Furthermore, PBS (0.1 mol/L, pH 7.4) solution served as a control, the absorbance value of the mixture was measured at 560 nm, and the Vc solution was employed as a positive control. The scavenging activity is expressed as:

$$Scavenging\;activity\left(\%\right)=\left(1-\frac{A_{1}-A_{2}}{A_{0}}\right)\times 100,$$

where A_0_ denotes the absorbance value of control (PBS instead of sample); A_1_ represents the absorbance value of the mixed sample; and A_2_ expresses the absorbance value of only sample without NBT (PBS instead of NBT).

#### 2.10.2 DPPH Radical Scavenging Activity

The DPPH radical scavenging activity of the GaE polysaccharide was measured using the method of Chen et al. with slight modification (Chen et al., 2019a). DPPH was dissolved in anhydrous ethanol to prepare 0.4 mmol/L solution. It was used right after it was ready. One milliliter of sample solutions was mixed with different concentrations (0.25–5.00 mg/L), and 1 ml of DPPH (0.4 mmol/L) solution was mixed. The solution was stirred and then incubated at a dark environment at 25°C for 30 min. The absorbance value of the mixture was measured at 517 nm, and the Vc solution served as a positive control. The scavenging activity is expressed as

$$Scavenging\;activity\left(\%\right)=\left(1-\frac{A_{1}-A_{2}}{A_{0}}\right)\times 100,$$

where A_0_ denotes the absorbance value of control (distilled water instead of sample); A_1_ is the absorbance value of the mixed sample; and A_2_ is the absorbance value of only sample without DPPH (anhydrous ethanol instead of DPPH).

#### 2.10.3 Hydroxyl Radical Scavenging Activity

The hydroxyl radical scavenging activity of the GaE polysaccharide was measured using the method reported by Qiao et al. with slight modification (Qiao et al., 2009). The GaE polysaccharide solution (0.5 ml) of different concentrations (0.25–5.00 mg/L) was mixed with 0.5 ml of salicylic acid solution (9 mmol/L), 0.5 ml of FeSO_4_ solution (9 mmol/L), and 0.5 ml of H_2_O_2_ solution (9 mmol/L). Subsequently, the mixture was stirred and incubated at 37°C for 40 min. The absorbance value of the mixture was measured at 510 nm, and the Vc solution was employed as a positive control. The scavenging activity is expressed as

$$Scavenging\;activity\left(\%\right)=\left(1-\frac{A_{1}-A_{2}}{A_{0}}\right)\times 100,$$

where A_0_ denotes the absorbance value of control (distilled water instead of sample and H_2_O_2_); A_1_ is the absorbance value of the mixed sample; and A_2_ represents the absorbance value of only sample without H_2_O_2_ (distilled water instead of H_2_O_2_ solution).

#### 2.10.4 ABTS Radical Scavenging Activity

The ABTS radical scavenging activity of the GaE polysaccharide was measured using the method described by Erel (2004) with minor modification. In brief, the reaction solution was prepared, 9.9 mg potassium persulfate was mixed with 15 ml of 5.55 mmol/L ABTS aqueous solution, stirred, and then incubated in a dark environment at 25°C for 15 h to form a blue-green solution. Subsequently, the mixture was diluted with PBS (100 μmol/L, pH 7.4) solution, and the absorbance value was determined at 734 nm. When the absorbance value of the mixture reached 0.70 ± 0.03, it could be employed for the GaE polysaccharide scavenging ABTS radial scavenging assay in 3 h. Afterward, the GaE polysaccharide samples of different concentrations (0.25–5.00 mg/L) were reacted with 0.2 and 8 ml ABTS solutions for 25 min, and then the absorbance value of the mixture was measured at 734 nm. The Vc solution served as a positive control. The scavenging activity is expressed as:

$$Scavenging\;activity\left(\%\right)=\left(1-\frac{A_{1}-A_{2}}{A_{0}}\right)\times 100,$$

where A_0_ denotes the absorbance value of control (distilled water instead of sample); A_1_ represents the absorbance value of the mixed sample; and A_2_ is the absorbance value of only sample without ABTS (distilled water instead of ABTS solution).

#### 2.10.5 Ferrous Ion-Chelating Ability

The ferrous ion-chelating activity of the GaE polysaccharide was measured using the method reported by Wang et al. with some modifications (Wang et al., 2012). First, 3 ml of distilled water was mixed with 75 μL of FeCl_2_ solution (2 mmol/L), and then 1.5 ml of the GaE polysaccharide solution with different concentrations (0.25–5.00 mg/L) was added. The mixed solution was maintained at 25°C for 3 min, and then 0.15 ml of ferrozine solution (5 mmol/L) was added. Subsequently, the mixture was shaken and incubated at 25°C for 10 min. The absorbance value of the mixture was measured at 562 nm, and the ethylenediaminetetraacetic acid (EDTA) solution was employed as a positive control. The chelating ability is expressed as:

$$Scavenging\;activity\left(\%\right)=\left(1-\frac{A_{1}-A_{2}}{A_{0}}\right)\times 100,$$

where A_0_ denotes the absorbance value of control (distilled water instead of sample); A_1_ represents the absorbance value of the mixed sample; and A_2_ expresses the absorbance value of only sample without FeCl_2_ (distilled water instead of FeCl_2_ solution).

#### 2.10.6 Reducing Power

The reducing power of the GaE polysaccharide was measured using the reported method of Yuan et al. with slight modified (Yuan et al., 2005). First, 1.2 ml of the GaE polysaccharide of different concentrations (0.25–5.00 mg/L) was mixed with 3 ml of phosphate buffer (0.2 mol/L, pH 6.6) and 3 ml of K_3_Fe(CN)_6_ solution (1%, w/v), and then it was placed in a 50°C environment and incubated for 20 min. After the reaction was complete, 1.2 ml of TCA solution (10%, w/v) was added, and then the mixture was centrifuged at 3,500 rpm for 10 min. Subsequently, 2 ml of supernatant was mixed with 2 ml of distilled water and 1.2 ml of FeCl_3_ (0.1%, w/v), and the absorbance of the mixture was measured at 700 nm after 10 min. The Vc solution served as a positive control. The reducing power is expressed as:

$$Reducing\;power=A_{1}-A_{0}$$

where A_0_ denotes the absorbance value of control (distilled water instead of FeCl_3_ solution) and A_1_ represents the absorbance value of the mixed sample.

### 2.11 Hypoglycemic Activity Assay Analysis

#### 2.11.1 *α*-Glucosidase Inhibitory Activity

The α-glucosidase inhibitory activity of the GaE polysaccharide was measured using the previous method with slight modification (Jia et al., 2017). First, 1.2 ml of the GaE polysaccharide of different concentrations (0.25–5.00 mg/L) was mixed with 75 μL of α-glucosidase solution (2 U/mL) and then incubated at 37°C for 10 min. Subsequently, 75 μL of pNPG (6 mmol/L) was added, and the mixture was incubated at 37°C for 20 min. After the reaction time was completed, 100 μL of Na_2_CO_3_ solution (1 mol/L) was added, and the reaction was terminated. The mixture was placed for 10 min, and the absorbance value was measured at 400 nm. The acarbose solution was employed as a positive control. The inhibition activity is expressed as:

$$Inhibitory\;activity\left(\%\right)=\left(1-\frac{A_{1}-A_{2}}{A_{0}}\right)\times 100,$$

where A_0_ denotes the absorbance value of mixture without sample (distilled water instead of sample solution); A_1_ represents the absorbance value of the mixed sample; and A_2_ expresses the absorbance value of mixture without α-glucosidase (distilled water instead of α-glucosidase solution)

#### 2.11.2 *α*-Amylase Inhibitory Activity

The α-amylase inhibitory activity of the GaE polysaccharide was measured using the method of Duan with slight modification (Duan et al., 2018). First, 0.2 ml of the GaE polysaccharide of different concentrations (0.25–5.00 mg/L) was mixed with 0.2 ml of phosphate buffer (0.2 mol/L, pH 6.9) and 0.2 ml of α-amylase solution (0.5 mg/ml). The mixture was incubated at 37°C for 10 min. Subsequently, 0.2 ml of soluble starch solution (1%, w/v) was added to the mixture for 10 min. After the reaction, 0.4 ml of DNS reagent was added to the mixture and kept in a 90°C water bath for 5 min. When the mixture was cooled to an ambient temperature, the absorbance value of the mixture was measured at 540 nm. The acarbose solution served as a positive control. The inhibition activity is expressed as:

$$Inhibitory\;activity\left(\%\right)=\left(1-\frac{A_{1}-A_{2}}{A_{0}}\right)\times 100,$$

where A_0_ denotes the absorbance value of mixture without sample (distilled water instead of the polysaccharide sample solution); A_1_ represents the absorbance value of the mixed sample; and A_2_ expresses the absorbance value of mixture without α-amylase (distilled water instead of α-amylase solution).

### 2.12 Statistical Analysis

The three repeated experiments with the respective group of data are expressed as mean ± standard deviation (SD) and were plotted using origin 2018. Analysis of variance (ANOVA) was conducted through Dunnett’s multiple-range tests (SPSS Statistics 24 software); *p*-value < 0.05 was considered statistically significant. To be specific, the IC_50_ values of radical scavenging and enzyme inhibitory activities by multiple comparisons of means were studied through the least significance difference (LSD) test of differences between the groups.

## 3 Results and Discussion

### 3.1 Chemical Composition of the GaE Polysaccharide

Table 1 lists the yields and chemical compositions of the GaE polysaccharides. The yield of GaE-G was found as the highest (7.82%), the yield of GaE-R was the lowest (4.21%), and GaE-B and GaE-Hyb were between them, which were 5.67% and 6.63%, respectively. The total sugar content of GaE-G was significantly higher than those of the other three groups (*p* < 0.05), and the content of GaE-R was found as the lowest (60.21%). Binding proteins and phenols could affect the functional properties exhibited by polysaccharides (Peng et al., 2012). Among the GaE polysaccharides, the content of binding proteins and total phenol of GaE-R was significantly higher than that of GaE-B, GaE-Hyb, and GaE-G, so GaE-R might exhibit stronger biological activity than the other three groups. Moreover, uronic acid, flavonoids, and sulfate radicals in polysaccharides had an effect on the biological activity exhibited by polysaccharides (Sun et al., 2008; Peng et al., 2012), whereas no uronic acid (detected by Blumenkrantz et al.’s method, Farhadi, 2017), flavonoids (Zhishen et al.’s method detection, Oliveira et al., 2016), and sulfate (Tabatabai’s method detection, Thaipong et al., 2006) were detected in the GaE polysaccharides. In brief, obvious differences were found in the chemical composition of the GaE polysaccharides, which might lead to differences in their biological activities.

**TABLE 1:** Yield and chemical compositions of four polysaccharides obtained by different varieties of *Gastrodia elata*.

Varieties	GaE-B	GaE-R	GaE-Hyb	GaE-G
Yield (%)^1^	5.67 ± 0.31^c^	4.21 ± 0.25^d^	6.63 ± 0.22^b^	7.82 ± 0.35^a^
Chemical composition (W%)^1^				
Total sugar	65.62 ± 1.24^b^	60.21 ± 0.96^c^	67.18 ± 1.82^b^	73.85 ± 2.36^a^
Protein	2.72 ± 0.18^c^	4.11 ± 0.29^a^	3.21 ± 0.11^b^	1.34 ± 0.08^d^
Total polyphenol	0.62 ± 0.004^b^	0.84 ± 0.006^a^	0.53 ± 0.003^c^	0.32 ± 0.002^d^

1 The values are presented as mean ± SD (standard deviation).

Values with a different letter of superscripts (a, b, c, and d) are significantly different (*p < 0.05*).

### 3.2 Monosaccharide Composition of the GaE Polysaccharide

**FIGURE 1:**
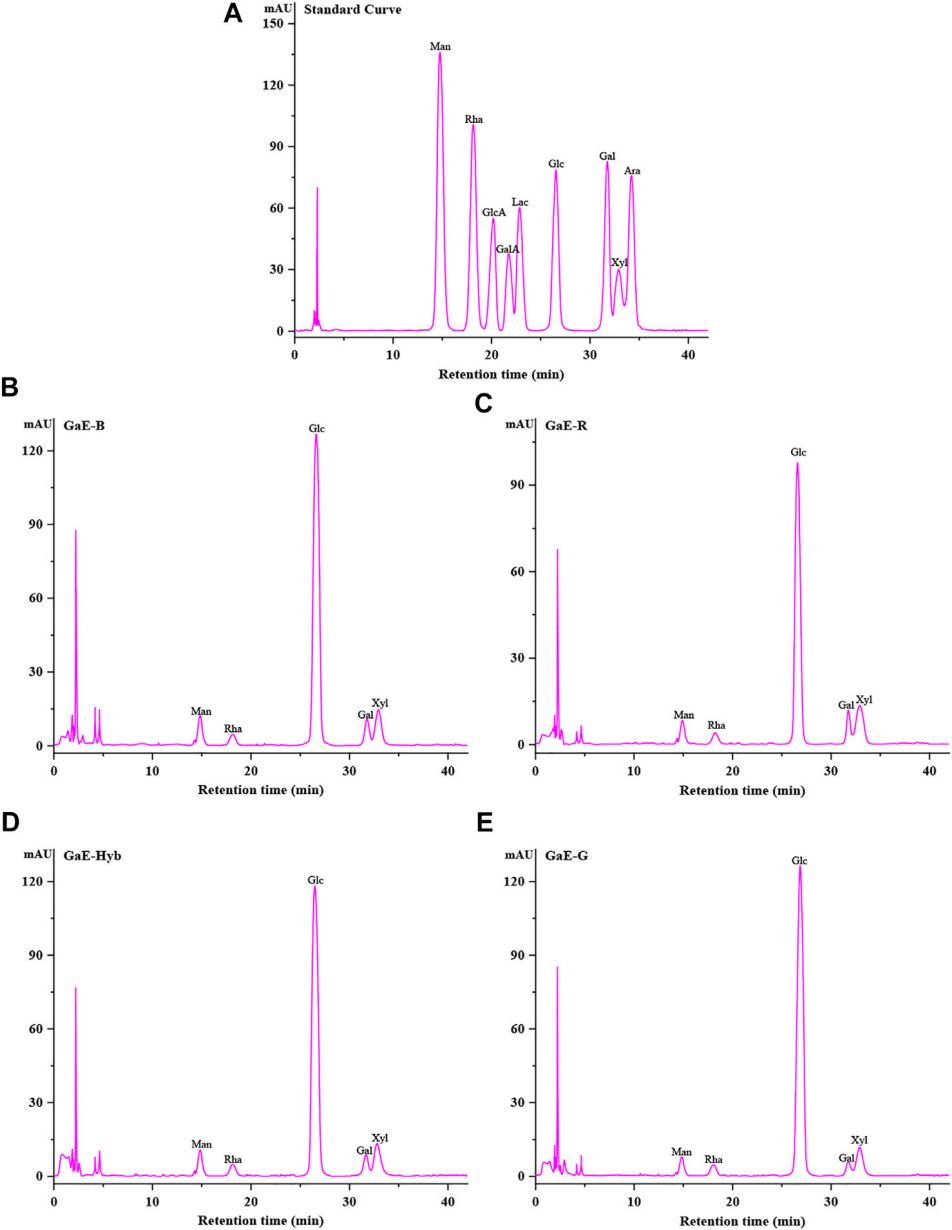
Monosaccharide composition of the polysaccharide with four different varieties of *Gastrodia elata*. **(A)** Standard curve; **(B)** GaE-B; **(C)** GaE-R; **(D)** GaE-Hyb; and **(E)** GaE-G.

The composition and content of monosaccharides often had an effect on the characteristic of polysaccharides. The monosaccharide composition of the GaE polysaccharides hydrolyzed after PMP derivatives were confirmed through comparison with the retention time of the HPLC chromatogram of the standard. As depicted in Table 2 and Figure 1, the GaE polysaccharides were heteropolysaccharides, and the GaE polysaccharides comprised mannose (Man), rhamnose (Rha), glucose (Glc), galactose (Gal), and xylose (Xyl). However, the content of each monosaccharide was different among various varieties. The GaE polysaccharides are primarily composed of glucose and xylose. To be specific, the content of glucose was found as the highest, higher than 50% in the respective group. The contents of GaE-B and GaE-Hyb glucose were close, and the content of glucose in GaE-G was found as the highest, reaching 81.88%, and the content of GaE-R was the lowest, only 71.01%. Second, the xylose content ranked second in the monosaccharide composition of the GaE polysaccharides. The GaE-R achieved the highest content of xylose, reaching 14.32%, and GaE-G achieved the lowest content of 8.4%, while the xylose contents of GaE-B and GaE were still close. It was found that the higher the content of Gal in polysaccharides, the stronger its antioxidant activity would be (Xing et al., 2005; Fan et al., 2014). Among the GaE polysaccharides, the Gal content in GaE-R was found as the highest at 6.41%, followed by GaE-B and GaE-Hyb, and the lowest was found as GaE-G, with a content of 3.11%. Accordingly, the content of Gal and Xyl might be one of the factors affecting the activity of the GaE polysaccharides.

**TABLE 2:** Monosaccharide composition of the *Gastrodia elata* polysaccharide with four different varieties.

Monosaccharide compositions (mol%)	GaE-B	GaE-R	GaE-Hyb	GaE-G
Mannose	5.36	5.07	4.83	3.64
Rhamnose	2.64	3.18	3.02	2.96
Glucose	77.35	71.01	77.58	81.88
Galactose	5.33	6.41	4.76	3.11
Xylose	9.34	14.32	9.81	8.40

### 3.3 Molecular Weight of the GaE Polysaccharide

**FIGURE 2:**
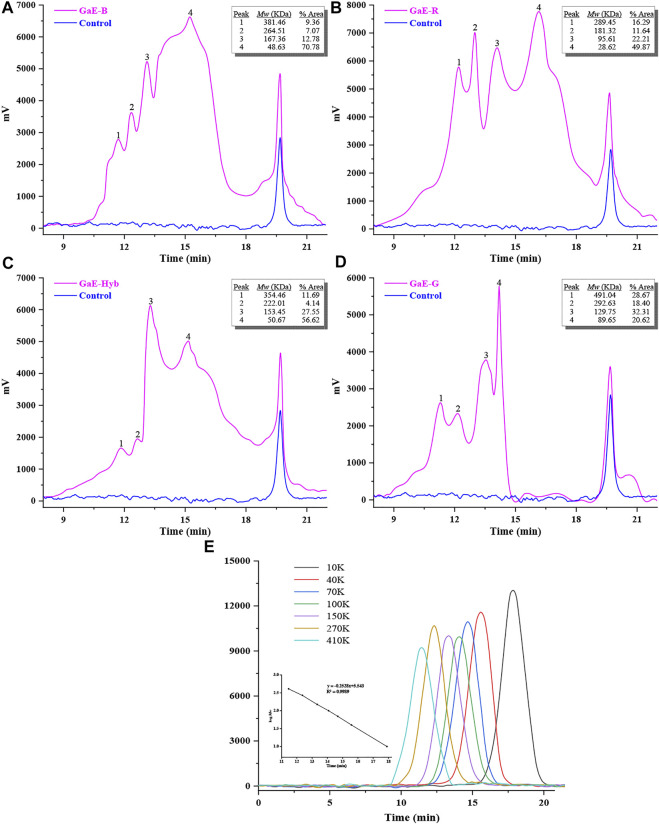
Molecular weight of the *Gastrodia elata* polysaccharide with four varieties. **(A)** GaE-B; **(B)** GaE-R; **(C)** GaE-Hyb; **(D)** GaE-G; and **(E)** standard curve.

As depicted in Figure 2, the HPGPC curves suggested that the GaE polysaccharides showed a wide molecular weight (*Mw*) distribution, which revealed that the GaE polysaccharides were heterogeneous polysaccharides, and they all had four main peaks. The molecular weights of the respective group arranged from large to small, and GaE-B was 381.46 KDa (9.36%, Peak1), 264.51 KDa (7.07%, Peak2), 167.36 KDa (12.78%, Peak3), and 48.63 KDa (70.78%, Peak4); GaE-R was 289.45 kDa (16.29%, peak1), 181.32 kDa (11.64%, peak2), 95.61 kDa (22.21%, peak3), and 28.6 kDa (49.87%, peak4); GaE-Hyb was 354.46 kDa (11.69%, peak1), 222.01 kDa (4.14%, peak2), 153.45 kDa (27.55%, peak3), and 50.67 kDa (56.62%, peak4); GaE-G was 491.04 kDa (28.67%, peak1), 292.63 kDa (18.40%, peak2), 129.751 kDa (32.31%, peak3), and 89.65 kDa (20.62%, peak4). As revealed by the comparison, the molecular weight of GaE-R was smaller than that of the other three groups, and the molecular weights of the four peaks were lower than 300 kDa. There was a slight difference in molecular weight between GaE-B and GaE-Hyb, whereas the content difference was significant. GaE-B low-molecular-weight polysaccharide content (70.78%) and GaE-Hyb intermediate-molecular-weight polysaccharide content were both higher. In comparison with the first three groups, the maximum molecular weight of GaE-G (491.04 kDa) was higher than that of the other three groups, the content was higher (28.67%), whereas the low-molecular-weight content (20.62%) was relatively lower. As compared with high-molecular-weight polysaccharides, the low-molecular-weight polysaccharides could more easily penetrate via the cell membrane, thus exhibiting higher physiological activities (Li et al., 2016; Zhang et al., 2016) (e.g., better ability to scavenge free radicals) (Cai et al., 2018). Some studies have found that polysaccharides with a molecular weight of 10–200 kDa usually showed better water solubility and biological activity (Chen and Kan, 2018; Chen et al., 2019b). Thus, in the further study, the biological activities of the GaEs polysaccharides will be assessed (e.g., antioxidant and hypoglycemic ability).

### 3.4 FT-IR Spectra of the GaE Polysaccharide

**FIGURE 3:**
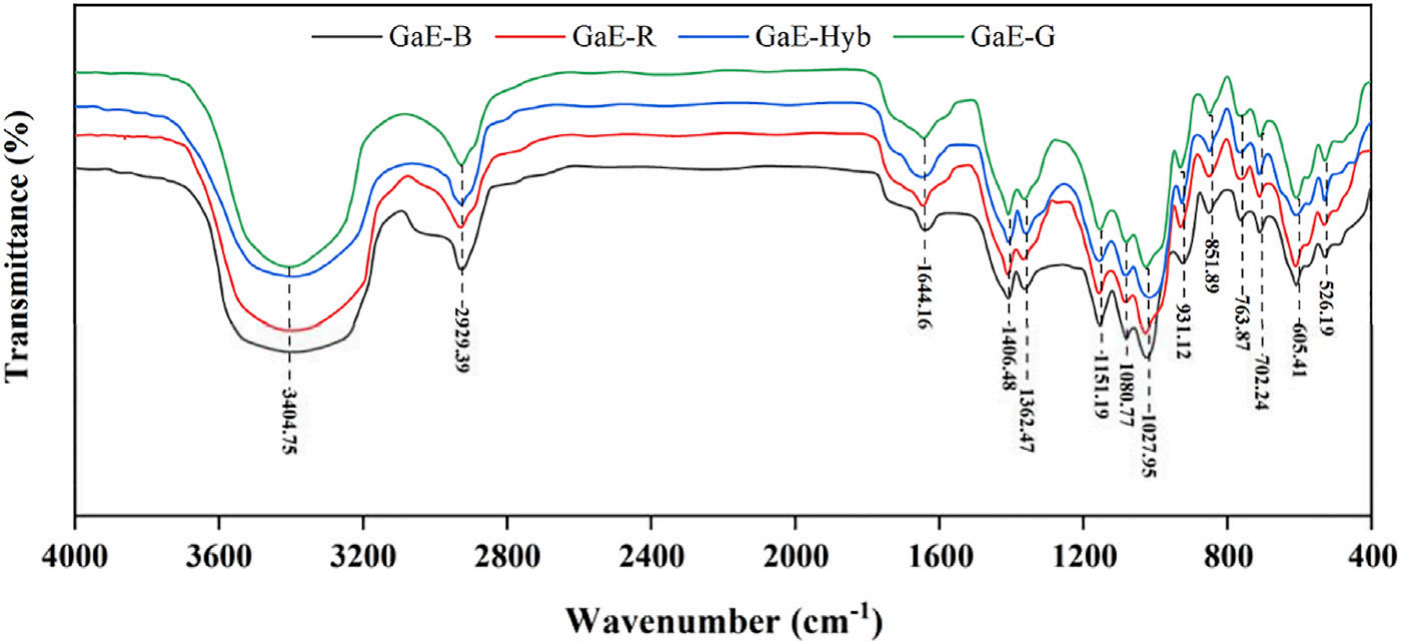
FT-IR spectra of the *Gastrodia elata* polysaccharides with four different varieties.

As depicted in Figure 3, no significant difference was identified in the FT-IR spectra of polysaccharides extracted from each of the GaEs, whereas there were typical signal peaks of polysaccharides. As revealed by the peak vibrating strongly close to 3400cm^−1^, there are stretching vibrations of O–H and N–H between molecules, or within the molecule (Zhang et al., 2013). The peak with a vibration close to 2925cm^−1^ suggested the existence of the stretching vibration of C–H between CH_3_, CH_2_, and CH (Yu et al., 2017), and the vibration peak close to 1640cm^−1^ was attributed to the asymmetric stretching vibration of C=O and C-O=O, which was the characteristic absorption peak of -CONH- (Zhang et al., 2013); the reason might be the existence of glycoprotein. The peak close to 1400 cm^−1^ indicated the existence of the C–H bending vibration, the vibration peak close to 1360 cm^−1^ indicates the existence of the O–H bending vibration, and the three peaks close to 900–1150 cm^−1^ indicated the existence of C–O–C and C–O–H of the pyranose ring bond asymmetric stretching vibration. To be specific, close to 1020 cm^−1^ was the C–O stretching vibration peak of sugar primary alcohols, and close to 1080 cm^−1^ was the C–O stretching vibration peak of sugar secondary alcohols (Yan et al., 2018). The peak close to 920 cm^−1^ indicated the existence of D-type glucopyranose asymmetric ring stretching vibration characteristic absorption (Farhadi, 2017), and the peaks close to 850 cm^−1^ indicate that the hemiacetal hydroxyl group of the GaE polysaccharides was largely α-configuration. As revealed by the FT-IR results, no significant difference was found in the spectra of the GaE polysaccharides, and they were found to contain similar principal components and chemical groups.

### 3.5 Thermal Characteristic of the GaE Polysaccharide

**FIGURE 4:**
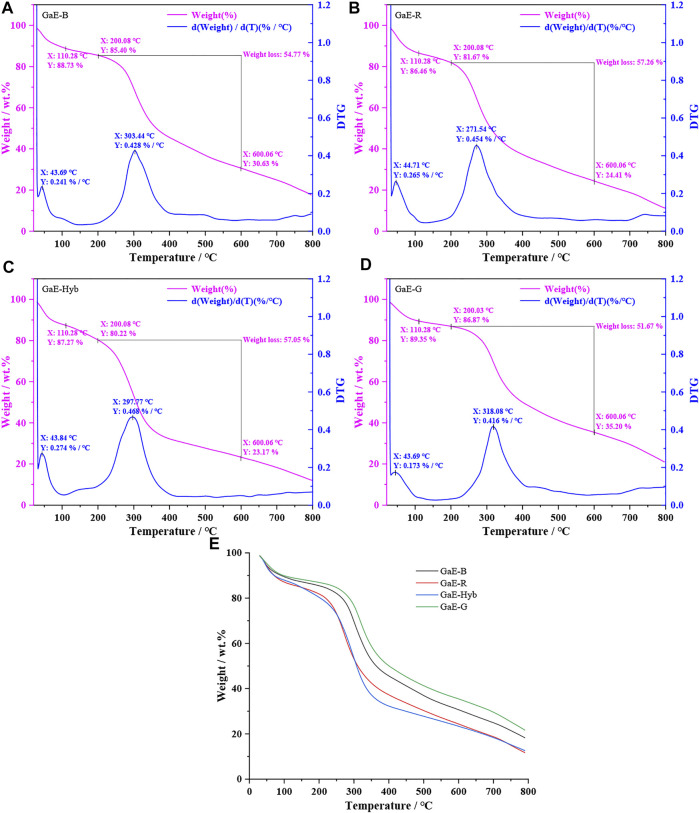
TGA thermogram of *Gastrodia elata* with four different varieties. **(A)** GaE-B; **(B)** GaE-R; **(C)** GaE-Hyb; **(D)** GaE-G; and **(E)** the four varieties of *Gastrodia elata*.

Thermogravimetric analysis (TGA) can indicate the thermal stability of a substance in a certain temperature range. The analysis of the thermal stability of polysaccharides can explore the correlation between the chemical composition of polysaccharides and other properties. As depicted in Figure 4, with the gradual increase of the temperature, the weight of the GaE polysaccharides tended to decrease, whereas the thermal decomposition curves of the GaEs were almost identical. The first weight loss occurred from 30°C to 110°C, primarily caused by the loss of free water and bound water of the GaE polysaccharides. When the temperature increased from 110°C to 200°C, the weight loss of GaE-Hyb was found as the highest and the GaE-G was the lowest. The second substantial weight loss was reported between 200°C and 600°C, largely due to the thermal melt degradation of the respective group of polysaccharides. The second degradation of the respective group’s weight loss exceeded 50%. In the second degradation temperature range, the weight loss of GaE-R and GaE-Hyb was higher than that of GaE-B and GaE-G, with GaE-R achieved the largest loss and GaE-G achieved the smallest loss. At a temperature of 600°C, the residual amount of the respective group of polysaccharides from high to low was GaE-G > GaE-B > GaE-R > GaE-Hyb, thus suggesting that the thermal stability of GaE-G was higher than that of the other three groups, probably correlated with the monosaccharide composition and molecular weight of GaE-G. At a test temperature of 800°C, the weight loss rate of the GaE polysaccharides continued to decrease, and the final residual content was 20.55% (GaE-G), 17.07% (GaE-B), 10.98% (GaE-R), and 12.52% (GaE-Hyb). As revealed by the results of thermogravimetric analysis, there might exist differences in structure, molecular composition, size, and other aspects of polysaccharides between the GaEs.

### 3.6 Particle Sizes and Zeta Potentials of the GaE Polysaccharide

**FIGURE 5:**
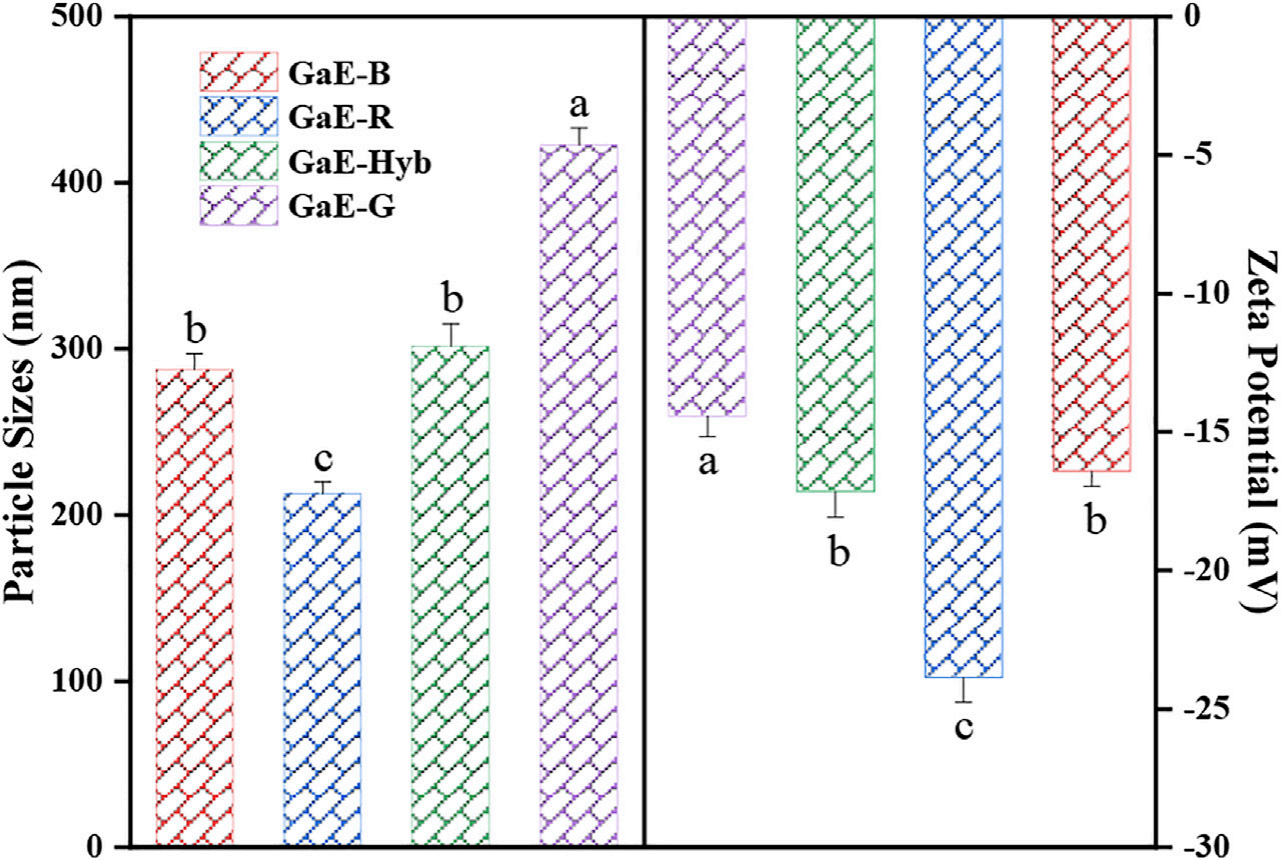
Particle sizes and zeta potentials of the *Gastrodia elata* polysaccharides with four different varieties.

The stability and solubility of polysaccharides can be assessed by investigating the particle size and zeta potential. Particle size refers to the hydrodynamic radius of molecules in the solution, and zeta potential is a measure strength of mutual repulsion or attraction between particles. The smaller the particle size, the higher the absolute value (positive or negative) of zeta potential would be, the larger the specific surface area of polysaccharide would be, the more uniform the dispersion in the solution would be, the higher the solubility would be, and the stability of the solution system would be (Dickinson, 2009). As depicted in Figure 5, the GaE polysaccharides were negatively charged, which suggested that the particles were mutually exclusive and difficult to aggregate into super molecules. The particle size of the four groups of polysaccharides showed a positive correlation with the molecular weight of the major components. GaE-G containing more macromolecules (491.04 kDa, 28.67%; 292.63 kDa, 18.40%) achieved the largest particle size (422.68 ± 10.11 nm). In contrast, GaE-R containing more small molecular weights (95.61 kDa, 22.21%; 28.6 kDa, 49.87%) achieved the smallest particle size (212.94 ± 7.22 nm). The absolute value of GaE-G zeta potential was found as the largest, which might arise from the small molecular weight, a considerable number of molecules of GaE-G, and more charged groups. As revealed by the abovementioned results, the size and stability of polysaccharide particles might account for the differences in the properties exhibited by the GaE polysaccharides in the four groups.

### 3.7 Scanning Electron Microscopy Images of *G. elata* Polysaccharide Microstructures

**FIGURE 6:**
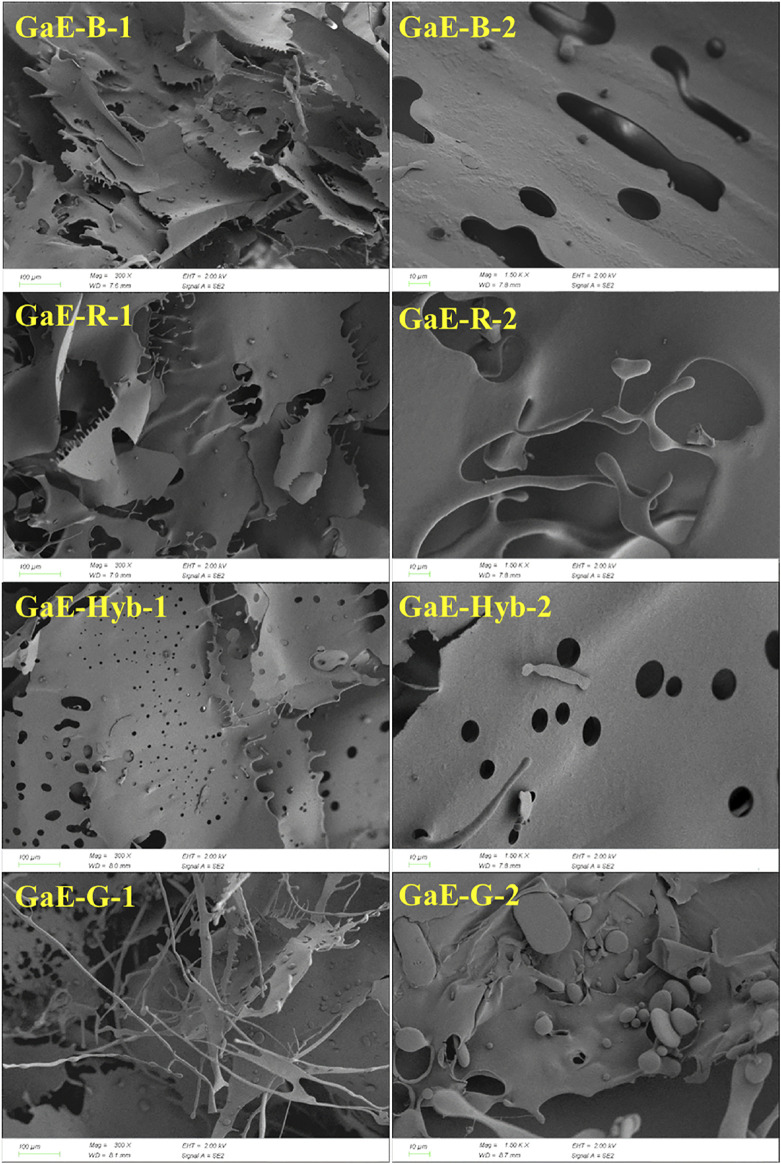
SEM photographs of polysaccharides obtained from different varieties of *Gastrodia elata* (1 for 300× and 2 for 1500×).

The bioactivity of polysaccharides is generally correlated with their three-dimensional (3D) structure. Figure 6. presents the microstructure of four varieties of *G. elata* polysaccharides. As depicted in the images, the 3D structure of each variety of *G. elata* showed certain differences. GaE-B, GaE-R, and GaE-Hyb were primarily sheet-like structures, while GaE-B and GaE-Hyb exhibited more pores and rough surfaces, which suggested that the 3D structure of the two was more similar. Although GaE-R also had a sheet-like structure, the surface was relatively smooth without small pores. GaE-G had a considerable number of particles and filamentous structures, and the 3D structure was relatively loose, which suggested that GaE-G polysaccharide particles might be mutually exclusive. As revealed by the biological activity of *G. elata* polysaccharide, the 3D structure of GaE-R could more significantly contribute to the improvement of polysaccharide activity.

### 3.8 Antioxidant Activity of the GaE Polysaccharide

#### 3.8.1 DPPH Radical Scavenging Activity

**FIGURE 7:**
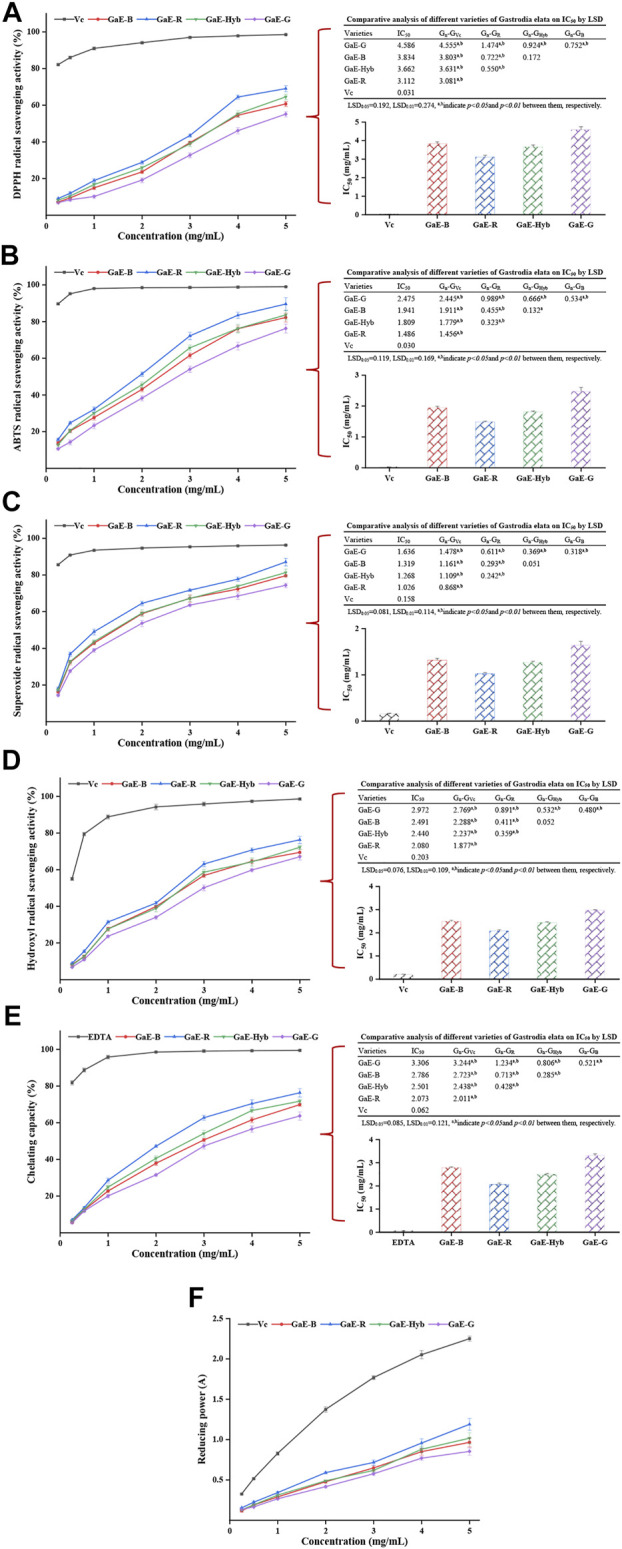
Antioxidant activities of the different varieties of *Gastrodia elata* polysaccharides at different concentrations *in vitro* and IC_50_ values: **(A)** DPPH radical scavenging activity and IC_50_ values; **(B)** ABTS radical scavenging activity and IC_50_ values; **(C)** superoxide radical scavenging activity and IC_50_ values; **(D)** hydroxyl radical scavenging activity and IC_50_ values; **(E)** chelating activity on ferrous ion and IC_50_ values; and **(F)** reducing power. The respective value is expressed as the means ± standard deviation (SD) of triplicate measurements.

DPPH refers to a stable nitrogen-centered free radical. Its solution has been commonly employed to measure the *in vitro* antioxidant capacity of a substance. The antioxidant activity of the substance can be assessed by measuring the change in absorbance (Oliveira et al., 2016). As depicted in Figure 7A, as the polysaccharide concentration increased, the DPPH free radical scavenging activity of the GaE polysaccharides was enhanced and dependent on concentration. GaE-R exhibited the higher scavenging activity than other varieties in the experimental concentration range, while the scavenging ability exhibited by GaE-G was always the weakest. Before the experimental concentration was 4 mg/ml, no significant difference was identified in the scavenging capacity of GaE-B and GaE-Hyb (*p* > 0.05), but the scavenging activity of the respective group was lower than that of the positive control group, vitamin C. The IC_50_ values of the respective group were obtained by curve fitting and found that the IC_50_ value of GaE-R was 3.112 mg/ml, which was the lowest in four varieties but still significantly higher than the positive control groups, vitamin C IC_50_ values (0.031 mg/ml). Through the LSD analysis, it was found that there were significant differences in IC_50_ values between GaE-R, GaE-G, and other groups, while no significant difference was found between GaE-B and GaE-Hyb. Accordingly, among the GaE polysaccharides, the DPPH scavenging ability of GaE-R was higher than other varieties.

#### 3.8.2 ABTS Radical Scavenging Activity

The ABTS radical scavenging method can be assessed for the antioxidant capacity of the substance by measuring the hydrophilic or lipophilic substances to scavenging the cationic free radical ABTs^+^ that cause changes in the absorbance of the system (Thaipong et al., 2006). As depicted in Figure 7B, with the increase in concentration, the ability of the GaE polysaccharides to scavenge ABT^+^ radicals gradually increased and showed concentration dependence. At the same experimental concentration, the scavenging ability of GaE-R was higher than that of the other groups, while the scavenging ability of GaE-G was the weakest, and no significant difference was identified between GaE-B and GaE-Hyb. By comparing the IC_50_ values of the respective group, it was found that GaE-R IC_50_ was the lowest (1.486 mg/ml) and GaE-G IC_50_ was the highest (2.475 mg/ml), while GaE-B and GaE-Hyb were somewhere in between but were higher than the IC_50_ values of vitamin C. Thus, among the four samples, the GaE-R has the strongest scavenging ability for ABT^+^ radicals, but it was still lower than that of vitamin C at the same concentration.

#### 3.8.3 Superoxide Radical Scavenging Activity

As a free radical produced in the process of biological metabolism, superoxide anion free radical can attack biological macromolecules, which are closely correlated to the body’s aging and pathological changes (Zhang et al., 2016). Thus, the determination of superoxide anion free radical scavenging ability can reflect the antioxidant activity of substances from another way. As depicted in Figure 7C, with the increase of polysaccharide concentration, the ability of the GaE polysaccharides to scavenge superoxide free radicals also gradually increased and showed the same concentration dependence as scavenging DPPH and ABT^+^ radicals. In the experimental concentration range, the scavenging capacity of GaE-R was always higher than that of other groups, and the difference was significant. No significant difference was found between GaE-B and GaE-Hyb but they were higher than that of GaE-G. When the concentration reached 0.5 mg/ml, the scavenging capacity of ascorbic acid increased sharply, and the scavenging ability was significantly different from the GaE polysaccharides. Among the IC_50_ values of superoxide radical scavenging by the GaE polysaccharides, GaE-R achieved the lowest IC_50_ values (1.026 mg/ml) and GaE-G achieved the highest IC_50_ values (1.636 mg/ml). Accordingly, the superoxide radical scavenging capacity of the GaE polysaccharides was ranked as GaE-R > (GaE-B and GaE-Hyb) > GaE-G, whereas the IC_50_ values of the four groups were higher than that of ascorbic acid (0.158 mg/ml).

#### 3.8.4 Hydroxyl Radical Scavenging Activity

Hydroxyl radical (OH) refers to a type of free radical with strong oxidation ability. It is capable of easily oxidizing various biological macromolecules with a high oxidation efficiency and fast reaction rate. It is an active oxygen free radical causing tissue lipid peroxidation, nucleic acid breaking, and protein and polysaccharide breakdown (Fan et al., 2014). As depicted in Figure 7D, with the increase in the polysaccharide concentration, the scavenging capacity of hydroxyl radicals in the respective group tended to increase, and the concentration dependence was indicated. At the experimental maximum concentration of 5 mg/ml, the scavenging capacity of GaE-R was higher than that of other groups, but no significant difference was identified among GaE-B, GaE-Hyb, and GaE-G. Comparing the IC_50_ values of the GaE polysaccharide samples, GaE-R had the lowest IC_50_ values (2.08 mg/ml), GaE-G had the highest IC_50_ values (2.08 mg/ml), GaE-B and GaE-Hyb have no significant difference, and no significant difference was found between GaE-B and GaE-HyB, whereas all of them were higher than that of ascorbic acid (0.203 mg/ml). As revealed by the results, among the GaE polysaccharides, GaE-R exhibited the strongest ability to scavenge hydroxyl free radicals.

#### 3.8.5 Ferrous Ion Chelating Capacity

Fe^2+^ refers to the active center of numerous oxidation reaction catalysts. In the body, Fe^2+^ is capable of reacting with O_2_ to generate superoxide radical and hydroxyl radical. When chelated, Fe^2+^ can reduce the generation of reactive oxygen species, thus indirectly playing a certain antioxidant role. As depicted in Figure 7E, the ferrous ion chelating capacity of each of the GaE polysaccharides tended to be enhanced as the concentration of the GaE polysaccharides increased in the experiment. The Fe^2+^ chelating capacity of GaE-R was the highest, and that of GaE-G was found as the weakest. The GaE polysaccharides’ chelating Fe^2+^ activity (y) was employed as a quadratic function of concentration (x) (*p* < 0.05), and the linear equation was yielded as y = −1.4749x2 + 20.897x + 1.794 (R^2^ = 0.9988) for GaE-B, y = −2.8178x2 + 29.131x+0.3111 (R^2^ = 0.9985) for GaE-R, y = −1.9059x2 + 23.657x + 1.3267 (R^2^ = 0.9985) for GaE-Hyb, and y = −1.1649x2 + 18.25x + 1.7959 (R^2^ = 0.997) for GaE-G. As revealed by the abovementioned quadratic fitting equations, the Fe^2+^ chelating activity of the GaE polysaccharides was dependent on concentration. Existing studies reported that the C=O functional group in the polysaccharide could form a cross-bridge with Fe^2+^ (Wang C. C. et al., 2010) to enhance the chelating ability of the polysaccharide to Fe^2+^. Compared with the other three groups, GaE-R exhibited a stronger chelating ability of Fe^2+^. The comprehensive comparison of the zeta potential, particle size, and molecular weight of the respective group suggested that GaE-R might contain more C = O functional groups since it formed a larger specific surface area in the solution and contained more molecular quantitative. As indicated by the comparison of the IC_50_ values of the Fe^2+^ chelating ability of the GaE polysaccharides, GaE-R achieved the lowest IC_50_ value (2.073 mg/ml), and the IC_50_ value of GaE-G was the highest (3.306 mg/ml), higher than those of EDTA in the positive control group. However, the GaE polysaccharides still had high practicability in chelating Fe^2+^ relative to polysaccharide macromolecules.

#### 3.8.6 Reducing Power

Iron reducing ability refers to the reaction of removing free radicals by reducing Fe^3+^ to Fe^2+^, and Fe^3+^ electrons are obtained from antioxidants (reducing agents). The reducing capacity and the antioxidant capacity were stronger, the latter of which has been commonly employed as an index to assess the antioxidant activity of polysaccharides. As depicted in Figure 7F, the reduction capacity of the GaE polysaccharides tended to be enhanced (the absorbance value gradually increased) as the experimental concentration of polysaccharides increased, which suggested that the antioxidant capacity of the GaE polysaccharides gradually increased as well. At the maximum experimental concentration of 5 mg/ml, the reducing ability of GaE-R was found as the strongest, that of GaE-G was the weakest, and that of GaE-B and GaE-Hyb were between GaE-R and GaE-G. However, the reducing ability of the GaE polysaccharides was lower than that of ascorbic acid in the positive control group at the same concentration. As revealed by the results, the GaE polysaccharides exhibited an electron-donating ability to scavenge free radicals and terminate the chain reaction of free radicals. To be specific, the Fe^3+^ reduction of GaE-R was slightly stronger than that of the other three groups, whereas there was slight difference in the Fe^3+^ reducing ability among the groups.

Excessive production of free radicals in the body can damage biofilms and biological macromolecules, accelerate aging, and cause diseases. In this study, the antioxidant activity of the GaE polysaccharides was obtained using a series of methods (e.g., DPPH radical scavenging ability, ABTS radical scavenging ability, superoxide radical scavenging ability, hydroxyl radical scavenging ability, Fe^2+^ chelating ability, and Fe^3+^ reducing ability). As revealed by the comparison of the antioxidant activity of the GaE polysaccharides, GaE-R was stronger than the other three groups in terms of DPPH radical, superoxide radical, hydroxyl radical, ABTS radical scavenging ability, Fe^3+^ reducing ability, and Fe^2+^ chelating ability; GaE-G exhibited the weakest capability relatively. Studies have shown that the antioxidant activity of polysaccharides was correlated with numerous factors (e.g., monosaccharide composition, molecular weight, the existence form and content of -C=O groups, the distribution of charge, and the contents of protein, sulfate, and phenols). The low quality polysaccharides exhibited higher free radical scavenging activity (Cai et al., 2018), which was probably because at the same concentration, low-molecular-weight polysaccharides contained more terminal hydroxyl and carbonyl groups, and a larger specific surface area was combined with free radicals in solution (Zhang et al., 2016). In this study, GaE-R had a lower molecular weight compared with the other three groups of the GaE polysaccharides, probably explaining why its biological activity was higher than the other groups. Second, the carboxyl and phenolic hydroxyl groups in proteins and polyphenols exhibited reducibility, which could enhance the antioxidant capacity of polysaccharides (Liao et al., 2014; Yan et al., 2018). GaE-R exhibited high free radical scavenging activity and reducing ability, which might be correlated with the factor that GaE-R contained more binding proteins and polyphenols. In addition, the composition and content of monosaccharides had an effect on the antioxidant activity of polysaccharides. In the abovementioned monosaccharides, more acidic sugars were contained, which significantly affected the free radical scavenging ability of polysaccharides since acidic sugars could more easily release H^+^ to be combined with reactive oxygen species (Yuan et al., 2019). Furthermore, existing research suggested that the lower the glucose content in the monosaccharides of polysaccharides, the higher the antioxidant activity of polysaccharides would be (Chen et al., 2010). In this study, the glucose content of GaE-R was found as the lowest and that of GaE-G was found as the highest, which might also account for the difference in antioxidant activities.

### 3.9 Hypoglycemic Activity *In Vitro*

#### 3.9.1 *α*-Glucosidase Inhibitory Activity *In Vitro*

**FIGURE 8:**
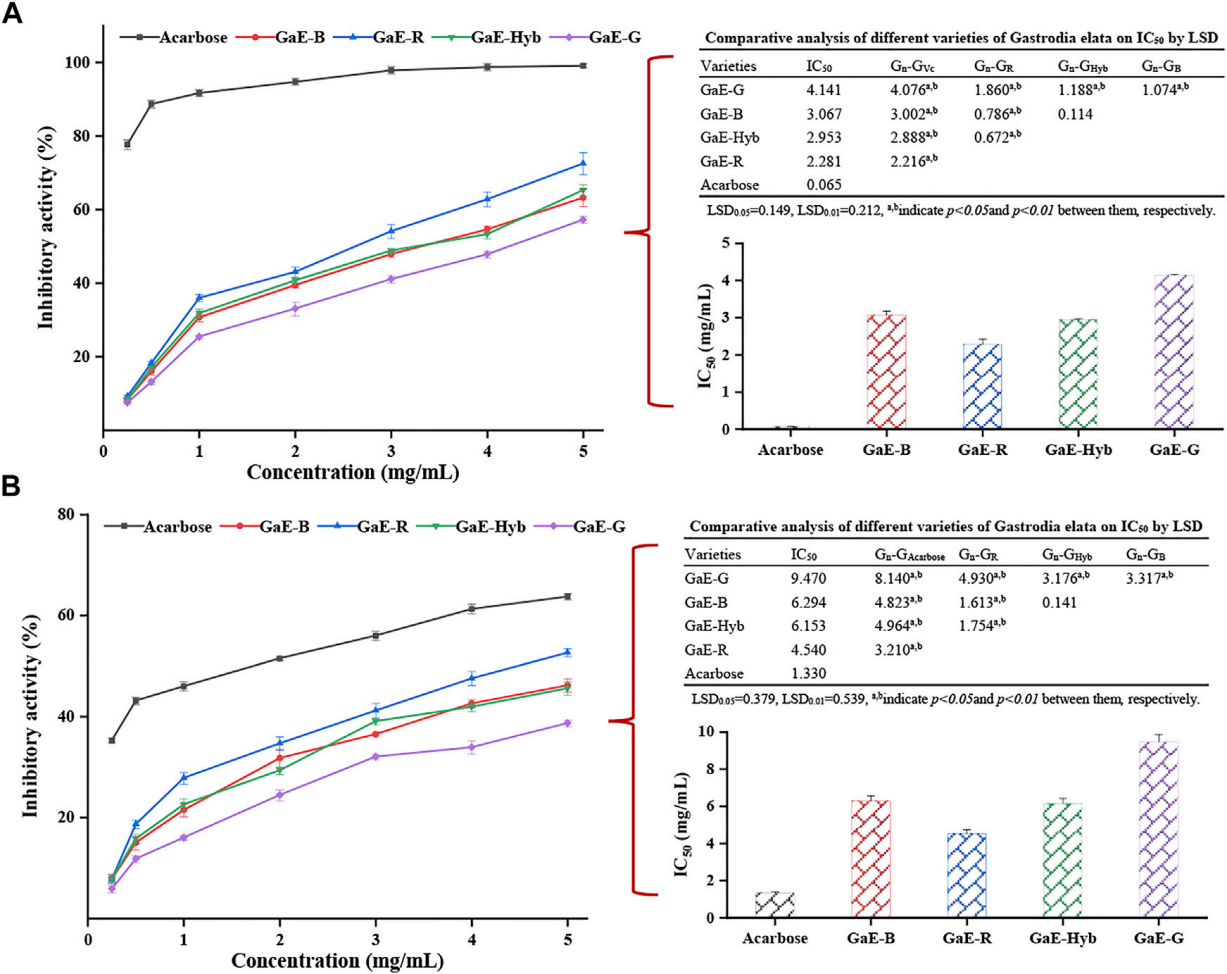
Hypoglycemic activities of the different varieties of *Gastrodia elata* polysaccharides at different concentrations *in vitro*: **(A)** α-amylase inhibitory activity and IC_50_ values and **(B)** α-glucosidase inhibitory activity and IC_50_ values. Each value is expressed as means ± standard deviation (SD) of triplicate measurements.

The body-ingested carbohydrates are largely hydrolyzed by α-amylase and α-glucosidase (Ali et al., 2006). If the activity of α-amylase and α-glucosidase can be inhibited, the blood sugar concentration in the body can be effectively reduced. Commonly used in clinic, α-amylase and α-glycosidase inhibitors include orlistat, acarbose, and miglitol (Kim et al., 1999), but taking the abovementioned inhibitors may cause damage to various viscera (Sall et al., 2014). However, natural macromolecular compounds inhibited enzyme activity more gently and had less toxic side effects than synthetic drugs (Bustanji et al., 2011). Plant polysaccharides can reduce blood glucose concentration by inhibiting the activity of related enzymes and promoting the secretion of insulin (Wang Y. et al., 2010). Thus, studying the α-amylase and α-glucosidase inhibitory activities of polysaccharides had practical significance for those patients suffering from hyperglycemia. As depicted in Figure 8A, with the increase in the concentration of the GaE polysaccharides, the inhibitory activity of the respective group to α-glucosidase also tended to increase and was dependent on concentration. In the experimental concentration range, the inhibitory activity of GaE-R was higher than that of other the GaE groups, and that of GaE-G was found as the lowest, which might be correlated with their size of molecular weight, whereas the inhibitory activity of the GaEs was significantly lower than that of the positive control of acarbose. The calculation of the IC_50_ value of the respective group after fitting curves suggested that the IC_50_ value of GaE-R was the lowest (2.281 mg/ml), and the IC_50_ value of GaE-G was the highest (4.14 mg/ml). No significant difference was identified between GaE-B and GaE-Hyb, whereas the IC_50_ value of the GaEs was significantly higher than the IC_50_ value of the positive control acarbose (0.065 mg/ml, *p* < 0.01). Nevertheless, the abovementioned natural polysaccharide macromolecules could still be sufficient to be developed and employed as hypoglycemic health food. In brief, the inhibitory activity of GaE-R on α-glucosidase was higher than that of the other three GaE polysaccharides.

#### 3.9.2 *α*-Amylase Inhibitory Activity *In Vitro*

Starch in food is primarily decomposed into glucose by α-amylase, which enters the blood to increase blood sugar concentration, inhibit the activity of α-amylase, and reduce the absorption of glucose by the body; as a result, the increase of blood sugar concentration in diabetic patients is delayed (Liu et al., 2017). In this study, the inhibitory activity of the GaE polysaccharides on α-amylase was investigated to explore the application value of the GaE polysaccharides in hypoglycemic drugs. As depicted in Figure 8B, the α-amylase inhibitory activity of the GaE polysaccharides in the respective group increased as the experimental concentration increased. In the concentration range of 2–3 mg/ml, the inhibition rates of GaE-R, GaE-B, and GaE-Hyb were close; in the concentration range of 4–5 mg/ml, the inhibition rate of GaE-R was higher than that of GaE-B and GaE-Hyb. The experimental concentration of 0.5–5 mg/ml suggested that the inhibition rate of GaE-G was significantly lower than that of other groups (*p* < 0.05), and almost no significant difference was found between GaE-B and GaE-Hyb. At the experimental concentration of 5 mg/ml, GaE-R exhibited the inhibitory activity of 52.62% ± 0.81%, the highest among the GaE polysaccharides, and the activity of GaE-G was found as the lowest at 38.63% ± 0.62%. The IC_50_ values of the GaE polysaccharides in the four groups were found as the lowest in GaE-R (4.54 mg/ml) and the highest in GaE-G (9.47 mg/ml), whereas the values were significantly higher than those of acarbose in the positive control group (1.33 mg/ml, *p* < 0.05). Although the value was lower than the positive control, relative to the natural biological macromolecules, it still could serve as a better α-amylase inhibitor. The hydroxyl or carboxyl groups in polysaccharides could be combined with amino acid residues in the active site of enzyme, thus competitively or non-competitively inhibiting the catalysis of the enzyme by the substrate (Wang et al., 2010 ; Wu et al., 2017).

In this study, the inhibitory activities of the GaE polysaccharides on enzymes were not exactly the same. Regardless of α-glucosidase or α-amylase, the inhibitory activity of GaE-R was higher than that of the other three groups, while no significant difference was found between GaE-B and GaE-Hyb (*p* > 0.05). In addition, GaE-G was always the lowest. It was found that the side-chain group of polysaccharides could be combined with the active site of enzyme, so as to reduce the catalytic activity of enzyme (Kim et al., 1999). Moreover, the charge on the polysaccharide could form a complex with α-glucosidase, thus reducing the activity of the enzyme (Jia et al., 2017). Accordingly, the differences in the activities of the respective group might arise from the different types and quantities of side-chain groups and charges of each of the GaE polysaccharide. In addition, the molecular weight and monosaccharide composition of polysaccharides might account for the abovementioned result (Zhang et al., 2010; Li et al., 2016; Chen et al., 2013). This is because the smaller the molecular weight, the more the side-chain groups would be at the same molar concentration, the more charges would carry, and the more binding to the active site of the enzyme would be. As revealed by the abovementioned results, the higher inhibitory activity of GaE-R than other groups might arise from the differences in molecular weight, structure, side-chain groups, and monosaccharide composition.

## 4 Conclusion

In this study, the characteristics of the GaE polysaccharides were investigated, which were largely described in accordance with structural characteristics, biological activity, and physicochemical properties. As revealed by the results, the GaE polysaccharides had different yields, chemical compositions, monosaccharide compositions, molecular weight distributions, physical properties, and biological activities, whereas most of the properties between GaE-B and GaE-Hyb were similar or showed no significant difference. The GaE polysaccharides were composed of the same type of monosaccharides, so they were heteropolysaccharides, and glucose was reported as the major component. However, differences in monosaccharide content were found among the varieties, and the GaE-R glucose content was found as the lowest. In general, GaE-R exhibited excellent antioxidant and glycosidase inhibitory activities *in vitro*, probably due to its lower molecular weight and glucose content, higher contents of binding protein, and polyphenols. In addition, the yield and total sugar content of GaE-R were lower than those of the other three varieties.

Although the GaE polysaccharides exhibited some physiological activities, the harvested *G. elata* polysaccharides would be impossible to extract and utilize, which is unacceptable in terms of cost. The efficacy of *G. elata*, a traditional Chinese medicine, could not be assessed only according to the physiological activity of polysaccharides, the content of the major medicinal components of *G. elata* (e.g., gastrodin, p-hydroxybenzyl alcohol, and p-hydroxybenzaldehyde) also directly affected the overall *G. elata* medicinal properties. This study only investigated the functions and properties exhibited by *G. elata* from the perspective of polysaccharide, which does not represent the overall medicinal value of *G. elata*. However, with the continuous expansion of the *G. elata* industry in China, *G. elata* has also been approved by the National Health Commission of the People’s Republic of China as a homologous of medicine and food. Consumers often stew or boil *G. elata* as a food supplement with other ingredients, which will disperse *G. elata* polysaccharide and other medicinal components into the soup, as an attempt to achieve a synergistic therapeutic effect between different types of activity of components. Moreover, the water extraction method was chosen to extract polysaccharides in this study because the water extraction method was closer to the method of obtaining *G. elata* polysaccharide in life. In brief, comparing the physicochemical properties and antioxidant activities of polysaccharides among different *G. elata* varieties can lay a relevant theoretical basis for enterprise processing or consumer purchase.

## Data Availability

The raw data supporting the conclusions of this article will be made available by the authors without undue reservation.
